# The Effect of Hydrostatic Pressure on Coronary Flow and Pressure-Based Indices of Coronary Stenosis Severity

**DOI:** 10.3390/jcm13061649

**Published:** 2024-03-13

**Authors:** Firas Al-Janabi, Grigoris V. Karamasis, Christopher M. Cook, Konstantinos Stathogiannis, Sarosh Khan, Samer Fawaz, Uzma Sajjad, Rohan Jagathesan, Paul R. Kelly, Reto A. Gamma, Kare H. Tang, Gerald J. Clesham, Thomas R. Keeble, John R. Davies

**Affiliations:** 1 Essex Cardiothoracic Centre, MSE Foundation Trust, Basildon SS16 5NL, UKgrigoris.karamasis@gmail.com (G.V.K.); kstathog@hotmail.com (K.S.); retogamma@nhs.net (R.A.G.); kare.tang1@nhs.net (K.H.T.); gerald.clesham@nhs.net (G.J.C.); john.davies32@nhs.net (J.R.D.); 2 Medical Technology Research Centre, Anglia Ruskin School of Medicine, Chelmsford CM1 1SQ, UK; 3 School of Medicine, National and Kapodistrian University of Athens, Attikon University Hospital, 12462 Athens, Greece

**Keywords:** FFR, iFR, coronary flow, hydrostatic pressure

## Abstract

**Background**: To assess whether hydrostatic pressure gradients caused by coronary height differences in supine versus prone positioning during invasive physiological stenosis assessment affect resting and hyperaemic pressure-based indices or coronary flow. **Methods**: Twenty-three coronary stenoses were assessed in twenty-one patients with stable coronary artery disease. All patients had a stenosis of at least 50% visually defined on previous coronary angiography. Pd/Pa, iFR, FFR, and coronary flow velocity (APV) measured using a Doppler were recorded across the same stenosis, with the patient in the prone position, followed by repeat measurements in the standard supine position. **Results**: When comparing prone to supine measurements in the same stenosis, in the LAD, there was a significant change in mean Pd/Pa of 0.08 ± 0.04 (*p* = 0.0006), in the iFR of 0.06 ± 0.07 (*p* = 0.02), and in the FFR of 0.09 ± 0.07 (*p* = 0.003). In the Cx, there was a change in mean Pd/Pa of 0.05 ± 0.04 (*p* = 0.009), iFR of 0.07 ± 0.04 (*p* = 0.01), and FFR of 0.05 ± 0.03 (*p* = 0.006). In the RCA, there was a change in Pd/Pa of 0.05 ± 0.04 (*p* = 0.032), iFR of 0.04 ± 0.05 (*p* = 0.19), and FFR of 0.04+−0.03 (*p* = 0.004). Resting and hyperaemic coronary flow did not change significantly (resting delta APV = 1.6 cm/s, *p* = 0.31; hyperaemic delta APV = 0.9 cm/s, *p* = 0.85). Finally, 36% of iFR measurements and 26% of FFR measurements were re-classified across an ischaemic threshold when prone and supine measurements were compared across the same stenosis. **Conclusions**: Pd/Pa, iFR, and FFR were affected by hydrostatic pressure variations caused by coronary height differences in prone versus supine positioning. Coronary flow did not change signifying a purely pressure-based phenomenon.

## 1. Introduction

Fractional flow reserve (FFR) and instantaneous wave-free ratio (iFR) are recommended for assessing the haemodynamic significance of coronary lesions and guide revascularisation [1]. They are defined as the ratio of distal coronary pressure (Pd) to aortic pressure (Pa) during hyperaemia (FFR), and during the “wave free period” of diastole (iFR) and both have binary cut-off points for determining coronary stenosis severity [2,3].

The reproducibility of these pressure-based indices has been shown [4], but the actual accuracy of intracoronary pressure measurements are influenced by various factors originating from the preparation (wire calibration, pressure equalization) or from the measurement itself (pressure damping, submaximal hyperaemia, distal wire “whipping”) [5]. Such a factor is the effect of the hydrostatic pressure created by the height difference between the distal position of the pressure wire sensor and the coronary ostium where pressure equalization took place. Coronary arteries lie in different vertical planes [6] and it has been shown that there is significant height variation between the proximal and distal vessels when the patient is lying flat (such as during coronary angiography and invasive physiological measurements) [7,8]. In clinical practice, this hydrostatic pressure effect is ignored, as the actual pressure gradient is usually small (<5 mmHg) [9]. Nevertheless, recent studies have suggested that the hydrostatic pressure gradient influences significantly pressure-based physiological indices [7,10,11,12,13,14,15], and as such the quantification of the hydrostatic impact on clinical practice remains poorly understood.

In this study, we studied the hydrostatic pressure effect in vivo by determining coronary pressure and flow across the same stenosis with standard supine and experimental prone patient positions. Our hypothesis was that the change in hydrostatic pressure associated with a change in patient position would impact hyperaemic and resting coronary pressure-based indices but not coronary flow measurements.

## 2. Methods

### 2.1. Study Design

This was a prospective cohort study in patients with stable angina admitted for pressure-based assessment of coronary stenosis at a single institution. All patients underwent standard supine measurements and experimental prone measurements. The inclusion criterion was patients with moderate coronary artery disease (>50% stenosis) on standard angiography. Exclusion criteria included inability to lie prone, contraindication to adenosine, recent acute coronary syndrome (<48 h), and severe renal dysfunction (eGFR < 30 mL/min/1.73 m^2^). The study was approved by the local ethics committees (East of England–Cambridge South Research Ethics Committee/REC reference: 17/EE/0003) and adhered to the principles of the Declaration of Helsinki. All participants gave written informed consent prior to participating in the study. The study was registered on ClinicalTrials.gov (identification code: NCT03097172).

### 2.2. Catheterisation Protocol

**Figure 1:**
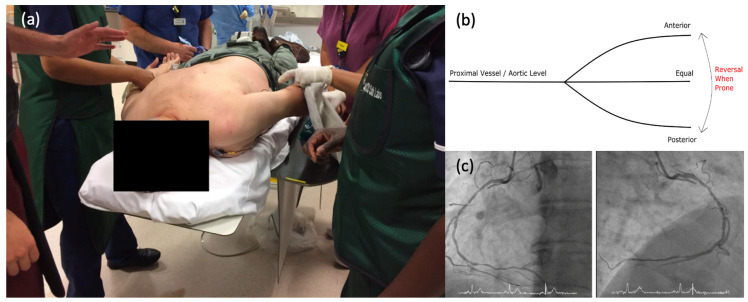
Positioning of the patient and acquired angiographic views. (**a**). Prone Positioning—Patient is positioned prone, with two members of the team on each side for safety. The left arm is draped and readied for arterial puncture. ECG leads are already attached. Permission for use was obtained from all involved. (**b**). Reversal of coronary arteries with an anterior or posterior course relative to proximal vessel. (**c**). Prone Angiographic Projection—Standard angiographic appearance of the RCA in a supine patient on the left. On the right, the RCA is seen in a prone patient with the origin appearing from the opposite side of the screen.

Patients were initially positioned prone on the catheterisation table and arterial access gained via the left radial artery. With the patient prone and the wrist pronated, the left hand/forearm laid at the typical right side of the catheterization table. The left radial artery was selected as the easiest and safest access point for both prone and supine positions. Then, the target vessel was intubated with a 6F guiding catheter. Intra-arterial unfractionated heparin (70–100 U/kg) and intracoronary nitroglycerin (500 mcg) were given prior to any measurements of coronary physiology. The optimal working view was determined, noting that coronary anatomy was reversed (horizontally) when the patient was positioned prone (Figure 1). A combined pressure and Doppler intracoronary guidewire (Combowire XT, Volcano Corporation, San Diego, CA, USA) was advanced to the tip of the guiding catheter for pressure normalisation. The guidewire was then advanced to the distal vessel. Slight adjustments in torque or forward/backward movement of the wire were made to achieve the best possible velocity Doppler trace. Once an optimal Doppler signal was obtained, the position of the wire was recorded on angiography acquisition to be used during the upcoming supine measurements. Resting Pd/Pa and peak coronary flow velocity (APV) were then measured across the stenosis. Hyperaemic Pd/Pa (i.e., FFR) and APV followed during steady state hyperaemia, with intravenous infusion of adenosine. The physiology guidewire was then retracted to the tip of the guide catheter to check for drift. After that, the physiology guidewire, the guiding catheter, and other equipment attached were removed from the patient. The sterile drape was removed with only the left radial sheath left in situ and the patient was turned to the standard supine position with the help of slide sheets and members of staff (two members on each side of the bed for extra safety). Extra care was taken for the radial sheath to remain sterile and in position; therefore, it was covered with a sterile drape during the turning-around manoeuvre. When the patient was in the supine position, she/he was re-draped/re-sterilized. Then, the guide catheter and physiology guidewire were re-inserted into the target vessel. The physiology guidewire was re-normalised and positioned in the exact location used during the prone measurements. All measurements were repeated. The supine FFR results were available to the operator to guide treatment. The study team did not have any further input and did not provide any guidance to the operators with regard to decision-making on the need for revascularisation or not. Supine measurements were deliberately taken secondarily, as it negated the need for a further turn of the patient should treatment be required.

### 2.3. Proximal Wire Position

In a subset of LAD lesions, FFR was measured during hyperaemic pullback, just distal to the stenosis (3 vessel diameters), rather than in the distal vessel. The aim was to assess whether hydrostatic effects were still as pronounced when the wire was positioned in a more proximal position.

### 2.4. Data Analysis

Pressure waveforms, coronary flow velocity, and electrocardiography data were directly extracted from the device console (Combomap V1.9, Volcano Corporation, San Diego, CA, USA) for further offline analysis. Data were analyzed offline using a custom software package designed with MATLAB version 6.0.0.88 (The MathWorks, Natick, MA, USA). FFR was calculated as the ratio of mean Pd to mean Pa across the whole cardiac cycle during hyperaemia. iFR was calculated as the mean Pd divided by the mean Pa during the wave-free period of diastole.

### 2.5. Change in Ischaemia Classification

The percentage of lesions crossing the ischaemic thresholds of Pd/Pa, iFR, and FFR were calculated by comparing prone and supine measurements. If an inferior artery position did not yield a higher pressure-based measurement, it was assigned a negative delta value.

### 2.6. Stenosis Severity

The stenosis severity percentage was calculated using quantitative coronary analysis (QCA), calibrated against the guiding catheter.

### 2.7. Statistical Analysis

Continuous variables were presented as means with standard deviation. Categorical variables were presented as numbers and percentages. Data from prone and supine positioning were compared for statistical significance using a student *t*-test for matched pairs. The sample size was calculated for 80% power, a 5% error rate, and a *p*-value of <0.05, using a paired *t*-test. The minimum difference expected between prone and supine physiology measurements using the mean FFR was 0.06. This calculated minimum difference was extrapolated from computed tomography coronary angiography data [4]. The minimum sample size was 6 per coronary artery, and 18 in total across all 3 major arteries. Significance was calculated at a *p*-value of <0.05. IBM SPSS^®^ statistics software (Version 25.0. IBM Corp., Armonk, NY, USA) was used.

## 3. Results

Eighty patients referred for pressure wire assessment were screened against the inclusion and exclusion criteria. Of the patients approached for recruitment after the initial screening process, three declined to participate in the study. Eventually, twenty-three coronary stenoses were analyzed in twenty-one patients. The baseline characteristics of the patients recruited are presented in Table 1. All patients were male; three female patients declined study participation. Ten LAD, seven Cx, and six RCA lesions were included in the analysis. In the RCA, the distal wire was placed in four cases in the PLV branch and two cases in the PDA branch. Two patients had two lesions assessed as part of one procedure: one with a lesion in the LAD and PDA and a second with a lesion in the PLV and Cx. All right coronary lesions were in dominant vessels. All pressure and velocity data collected were assessed and deemed of good quality. Pressure traces used were without drift or pressure dampening. In one patient, iFR could not be calculated offline.

One patient suffered coronary dissection in a side branch of the main vessel related to physiology wire manipulation. This was treated conservatively with no clinical sequelae and the patient was discharged home the same day. There were no other adverse events post-procedure for any recruited patient. There was no infection or stroke complication associated with the study protocol.

### 3.1. Pressure-Based Indices of Stenosis Severity

#### 3.1.1. Pd/Pa

**Figure 2:**
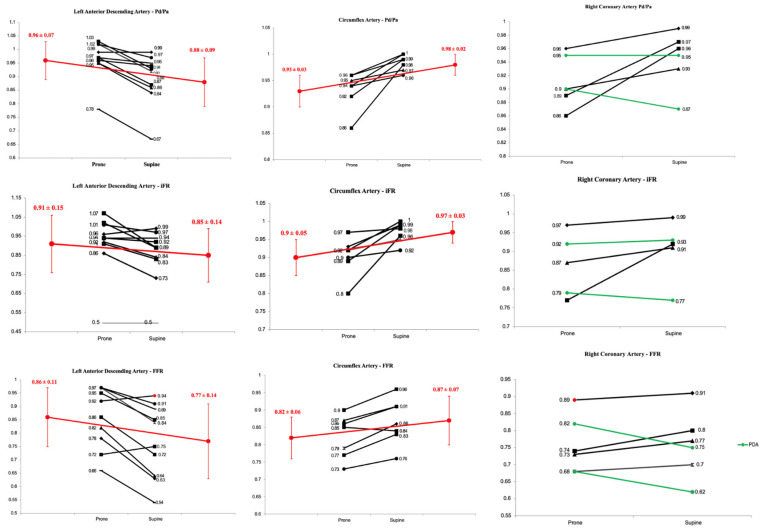
Changes in pressure-based indices in the prone and supine positions. Black lines signify individual changes, green lines individual changes in PDA and red lines changes in mean values.

Prone versus supine data for all Pd/Pa measurements are presented in Table 2, and individual changes per vessel are in Figure 2. In the LAD, there was a significant change of 0.08 ± 0.04 between prone and supine measurements (0.96 ± 0.07 vs. 0.88 ± 0.09, *p* = 0.0006). In the Cx, there was a significant change of 0.05 ± 0.04 between prone and supine measurements (0.93 ± 0.03 vs. 0.98 ± 0.02, *p* = 0.009). In the RCA–PDA, prone vs. supine Pd/Pa was 0.93 ± 0.03 vs. 0.91 ± 0.06, and in the RCA–PLV, it was 0.91 ± 0.07 vs. 0.98 ± 0.02 (*p* = 0.32).

#### 3.1.2. Instantaneous Wave Free Ratio (iFR)

Prone versus supine data from all iFR measurements - presented in Table 3, and individual changes per vessel are in Figure 2 In the LAD, prone vs. supine iFR was 0.91 ± 0.16 vs. 0.85 ± 0.14 (delta change 0.06 ± 0.07, *p* = 0.02). In the Cx, prone vs. supine iFR was 0.90 ± 0.05 vs. 0.97 ± 0.03 (delta change 0.07 ± 0.04, *p* = 0.01). In the RCA-PDA, prone vs. supine iFR was 0.86 ± 0.09 vs. 0.85 ± 0.11 and in the RCA-PLV, it was 0.87 ± 10 vs. 0.93 ± 0.07 (*p* = 0.19).

#### 3.1.3. Fractional Flow Reserve (FFR)

Prone versus supine data from all FFR measurements are presented in Table 4, and individual changes per vessel are in Figure 2. In the LAD, prone vs. supine FFR was 0.86 ± 0.11 vs. 0.77 ± 0.14 (delta change 0.09 ± 0.07 *p* = 0.003). In the Cx, prone vs. supine FFR was 0.82 ± 0.06 vs. 0.87 ± 0.07 (delta change 0.05 ± 0.03, *p* = 0.006). In the RCA–PDA, prone vs. supine FFR 0.75 ± 0.10 vs. 0.69 ± 0.10 and in the RCA–PLV, it was 0.82 ± 0.10 vs. 0.86 ± 0.08 (*p* = 0.004).

### 3.2. Proximal Wire Position

**Figure 3:**
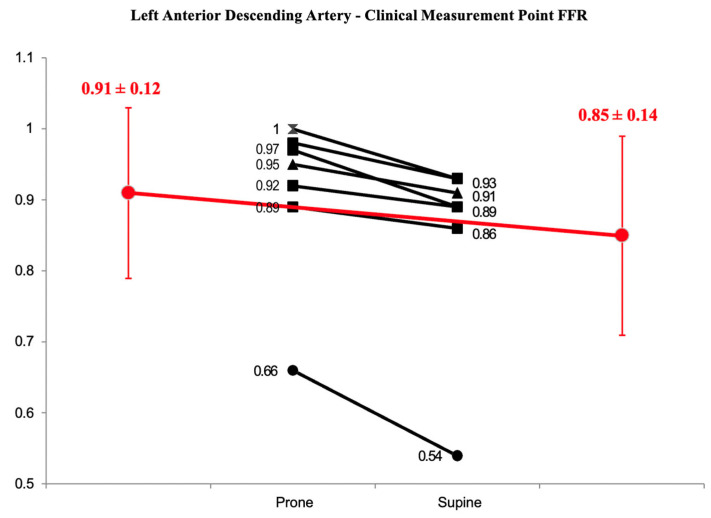
Prone vs. supine FFR in the LAD with the distal wire placed at a clinical measurement point. Mean values are in red. The difference is statistically significant (*p* < 0.05).

For a subset of 7 LAD lesions, the wire was not placed at the very distal vessel, but instead, it was placed distal to the stenosis as it would have been in usual clinical practice. For these measurements, the prone mean FFR was 0.91 ± 0.12 and the supine mean FFR was 0.85 ± 0.14 (delta change of 0.06 ± 0.04, *p* = 0.003) (Figure 3).

### 3.3. Stenosis Re-Classification

Patient position change led to eight patients crossing the ischaemia threshold for iFR (36%) and six patients crossing the FFR threshold (26%) (Table 5).

### 3.4. Doppler Flow Measurements

**Figure 4:**
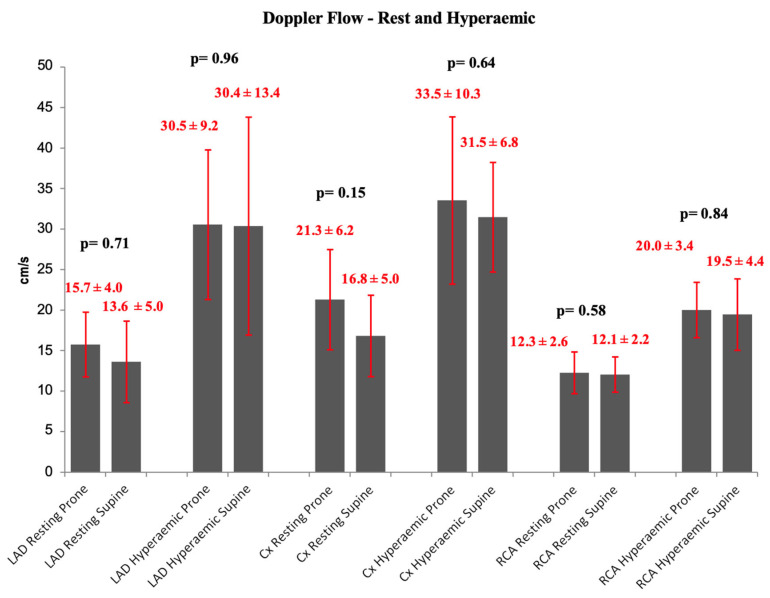
Resting and hyperaemic Doppler coronary flow per artery—prone vs. supine ± SD. There are no statistically significant differences.

There were no statistically significant differences in Doppler flow between superior and inferior positions in the resting or hyperaemic states (Figure 4).

## 4. Discussion

This is the first study to perform and compare coronary pressure and flow measurements in humans in the standard supine and experimental prone patient positions. The main study findings were:

1. There was a statistically significant difference in pressure-based indices across the same stenosis when comparing prone to supine patient measurements.
2. The inferior artery position (i.e., Cx and RCA–PLV) produced statistically higher Pd/Pa, iFR, and FFR values compared to the superior artery position (i.e., LAD and RCA–PDA).
3. Using a binary cut-off, approximately one-third of iFR and one-fourth of FFR measurements were re-classified across an ischaemic threshold due to a change in patient position.
4. Doppler flow velocity did not change when prone and supine positions were compared.

Pressure-based coronary indices are used to assess the severity of coronary stenosis. Pressure is used as a surrogate for flow, largely due to practical reasons. However, despite the ease of use, pressure-based indices have some confounding factors; hydrostatic pressure is among these. When FFR or iFR is measured, there is the assumption that both distal and proximal pressures are at the same level in the heart relative to the pressure transducer [5]. However, when the wire is advanced at the distal vessel, the distal sensor is seldom at the same vertical point as the proximal sensor. In a supine patient, anterior vessels, such as the left anterior descending artery (LAD) and posterior descending artery (PDA) take a superior course relative to the coronary ostium and posterior vessels, such as the circumflex artery (Cx) and the posterior left ventricular artery (PLVA) take an inferior course [7,8]. The vertical distance between the distal and proximal pressure sensor creates a pressure difference between these two points, caused by the hydrostatic effect. The magnitude of this is approximately 0.8 mmHg/cm, increasing pressure inferiorly and reducing it superiorly [8]. 

The effect of the hydrostatic gradient is evident in clinical practice, for example when a value of FFR > 1.0 is noted and is not caused by drift. As described in a large physiological registry, this predominantly occurs in the LCx, a vessel with an inferior course [16]. Furthermore, the hydrostatic effect could be one of the reasons (along with supplied myocardial mass, vessel tapering, and diffuse atherosclerosis) that FFR or non-hyperaemic pressure ratios in LAD are consistently lower than in other vessels [17,18,19]. However, the impact of the hydrostatic pressure gradient is not counted for physiological measurements as it is considered small and as such not clinically relevant. Indeed, in normal coronary anatomy, the hydrostatic effect is usually <5 mmHg [9]. This small pressure gradient is expected to influence more non-hyperaemic pressure ratios like iFR as the observed gradients across stenosis are smaller compared to FFR. A transtenotic gradient of approximately 10 mmHg is required to class a lesion as significant using a resting index [3]. A change of 5 mmHg for example accounts for 50% of the required gradient. FFR requires a larger transtenotic gradient for significance (20 mmHg), meaning a change of 5 mmHg is of less relative importance (accounting for 25% of the transtenotic gradient). Indeed, in our study, more patients were reclassified based on iFR measurements than on FFR measurements.

A few recent studies have suggested that hydrostatic pressure is a relevant factor influencing FFR or non-hyperaemic pressure ratio measurements. Harle et al. used a dynamic flow simulator to perform in vitro measurements and showed a correlation between absolute pressure differences and coronary height differences [8]. Other studies used recorded pressure measurements and compared the actual pressure ratios with pressure ratios corrected for hydrostatic pressure showing a reclassification of 12–13% for FFR and 20–27% for non-hyperaemic pressure ratios [13,14,15]. One study used an animal model and took FFR measurements during various height differences between the guide catheter tip and the distal pressure wire sensor produced by body rotation (20° to the right and 25° to the left) with or without vertical inclination (10° above and 10° below) of the catheterisation bed [12]. Two studies in humans took FFR and non-hyperaemic pressure ratio measurements in supine, prone [11], or left and right lateral [10] positions. All three experimental studies showed that adjustment for hydrostatic pressure increased pressure-derived values in anterior vessels and decreased values in posterior vessels, leading to similar rates of stenosis severity reclassification. The results of our study are consistent with those of previous studies. Our study is unique as it is the only one where FFR, iFR, and actual Doppler flow measurements were performed. Flow measurements did not significantly change, supporting the concept that change in pressure-based indices appears to be purely a pressure-based phenomenon rather than a change in coronary flow velocity.

It is appreciated that in clinical practice, the physiology wire may not be as distal as stipulated in this study protocol. The hydrostatic effect is more pronounced as the measurement point becomes progressively distal. As such, there may be a lessened hydrostatic effect in a ‘real world’ situation. In the subset of LAD stenoses, however, with the wire in a more proximal ‘clinical’ position, a significant difference was still observed between prone and supine positioning. It appears, therefore, that even with a submaximal hydrostatic effect, a statistically significant difference remains. In a distal stenosis, especially but not exclusively in the LAD, clinicians should be mindful of the potential effects of hydrostatic pressure. Importantly, the very distal pressure values become more relevant in the current era of CT and angiographically derived FFR, where full coronary tree vessel physiology value “mapping” is provided by commercially available vendors. 

The value of this study is that it emphasizes that physiological indices should not be approached as dichotomous values for justifying or deferring revascularisation. Treatment strategy is not a binary process, and clinical judgment should be used to combine multiple factors. The hydrostatic effect may be a further factor the operator should be mindful of when deciding on a final treatment strategy. In this direction, it is important to note that in the largest study correcting for measured pressure traces for hydrostatic pressure, when the grey zone of the different indices was considered, there was almost zero reclassification regarding functional significance (0.17%) [15]. The grey zones used in this study were 0.75–0.80 for FFR and 0.86–0.93 for non-hyperaemic pressure ratios. The use of grey zones underlines the fact that values around the cut-off points should be interpreted using clinical judgement in the context of the patient’s clinical presentation and symptoms and clinical and anatomical factors, etc. Importantly, similar to our study, the impact of the hydrostatic pressure on functional lesion severity was more prominent in the non-hyperaemic pressure ratios compared with the FFR.

Finally, it may also be possible to adapt current coronary technologies to abolish the hydrostatic effect. A newly available pressure guidewire (Wirecath Cavis Technologies AB, Uppsala, Sweden) is not affected by hydrostatic pressure due to its open-wire technology (a waterfilled interior and an external pressure transducer for hemodynamic measurements) [6]. This new guidewire is currently being studied in the ongoing PWCOMPARE study (NCT04802681) [15]. The study will provide simultaneous FFR measurements with different pressure wires assessing the extent and importance of hydrostatic pressure on these measurements. 

This study has several limitations. It consisted of a small number of patients, despite meeting the required power for analysis. Although all patients were male, it has been shown that the male sex correlates with larger vertical distance measurements of the vessel to the aorta [8]. The distal placement of the wire in the artery of interest may not always be mirrored in clinical practice, leading to an overestimation of the hydrostatic effect in all but distal lesions. Importantly, in this study, the height difference between the catheter tip and distal wire position is inverse between the prone and supine positions, but not neutralised. Therefore, the reclassification percentage for prone versus supine pressure ratios reported is probably larger than in reality. Venous pressure cannot be safely measured in a prone patient, thus it was not included in the FFR calculation.

## 5. Conclusions

In this study, coronary pressure and flow measurements were performed for the first time in the standard supine and experimental prone patient positions. The study showed that pressure-based indices across the same stenosis were increased for anterior vessels and decreased for posterior vessels. However, coronary flow did not change, signifying a purely pressure-based phenomenon. The operators should be mindful of the hydrostatic pressure gradient effect on pressure-based indices, appreciate the limitations of dichotomous cut-off values, and interpret physiological measurements using clinical judgement, considering clinical and anatomical factors. 

## Figures and Tables

**Table 1:** Patient baseline characteristics.

	(%)
Total Number	23 (100)
Age	63 ± 7
Male	23 (100)
Height (cm)	174 ± 18
Smoking	1 (4)
Diabetes	3 (13)
Hypertension	10 (43)
Hypercholesterolaemia	11 (48)
Family History of CAD	2 (9)
Ejection Fraction	52 ± 3

**Table 2:** Prone and supine mean Pd/Pa with delta changes.

Vessel (*n*)	Prone Pd/Pa Mean (±SD)	Supine Pd/Pa Mean (±SD)	Delta Change Mean (±SD)	*p*-Value
LAD (10)	0.96 (0.07)	0.88 (0.09)	0.08 (0.04)	0.0006
Circumflex (7)	0.93 (0.03)	0.98 (0.02)	0.05 (0.04)	0.009
RCA–PDA (2)	0.93 (0.03)	0.91 (0.06)	0.02 (0.02)	
RCA–PLV (4)	0.91 (0.07)	0.98 (0.02)	0.07 (0.05)	0.032
All (±SD) (23)	-	-	0.05 (0.04)	<0.0001

SD = Standard deviation.

**Table 3:** Prone vs. supine iFR and delta changes.

Vessel (*n*)	Prone iFR Mean (±SD)	Supine iFR Mean (±SD)	Delta Change Mean (±SD)	*p*-Value
LAD (10)	0.91 (0.16)	0.85 (0.14)	0.06 (0.07)	0.02
Circumflex (7)	0.90 (0.05)	0.97 (0.03)	0.07 (0.04)	0.01
RCA-PDA (2)	0.86 (0.09)	0.85(0.11)	0.01 (0.02)	
RCA-PLV (3)	0.87 (0.10)	0.94 (0.04)	0.07 (0.07)	0.19
All (±SD) (22)	-	-	0.06 (0.05)	<0.0001

**Table 4:** Prone vs. supine FFR.

Vessel (*n*)	Prone FFR Mean (±SD)	Supine FFR Mean (±SD)	Delta Change Mean (±SD)	*p*-Value
LAD (10)	0.86 (0.11)	0.77 (0.14)	0.09 (0.07)	0.003
Circumflex (7)	0.82 (0.06)	0.87 (0.07)	0.05 (0.03)	0.006
RCA–PDA (2)	0.75 (0.10)	0.69(0.10)	0.06 (0.02)	
RCA–PLV (4)	0.82 (0.10)	0.86 (0.08)	0.04 (0.03)	0.004
All (±SD) (23)	-	-	0.06 (0.04)	<0.0001

**Table 5:** Restenosis classifications based on binary cut-offs of 0.89 and 0.80 for iFR and FFR, respectively.

Index	Total Number	N Crossing Threshold	% Crossing Threshold
iFR	22	8	36%
FFR	23	6	26%

## Data Availability

Data are available on demand.
